# Genetic Analysis of Human Chymotrypsin-Like Elastases 3A and 3B (CELA3A and CELA3B) to Assess the Role of Complex Formation between Proelastases and Procarboxypeptidases in Chronic Pancreatitis

**DOI:** 10.3390/ijms17122148

**Published:** 2016-12-20

**Authors:** Andrea Párniczky, Eszter Hegyi, Anna Zsófia Tóth, Ákos Szücs, Andrea Szentesi, Áron Vincze, Ferenc Izbéki, Balázs Csaba Németh, Péter Hegyi, Miklós Sahin-Tóth

**Affiliations:** 1 Center for Exocrine Disorders, Department of Molecular and Cell Biology, Boston University Henry M. Goldman School of Dental Medicine, Boston, MA 02118, USA; andrea.parniczky@gmail.com (A.P.); heszter@bu.edu (E.H.); tothannadr@gmail.com (A.Z.T.); 2 First Department of Surgery, Semmelweis University, 1082 Budapest, Hungary; szucs.akos@gmail.com; 3 Institute for Translational Medicine, University of Pécs, 7624 Pécs, Hungary; szentesiai@gmail.com (A.S.); hegyi2009@gmail.com (P.H.); 4 First Department of Medicine, University of Szeged, 6720 Szeged, Hungary; nemeth.balazs@med.u-szeged.hu; 5 First Department of Medicine, University of Pécs, 7624 Pécs, Hungary; vincze.aron@pte.hu; 6 Department of Gastroenterology, St. George Teaching Hospital of County Fejér, 8000 Székesfehérvár, Hungary; fizbeki@gmail.com

**Keywords:** pancreas, chronic pancreatitis, digestive protease, zymogen complexes, elastase

## Abstract

Human chymotrypsin-like elastases 3A and 3B (CELA3A and CELA3B) are the products of gene duplication and share 92% identity in their primary structure. CELA3B forms stable complexes with procarboxypeptidases A1 and A2 whereas CELA3A binds poorly due to the evolutionary substitution of Ala241 with Gly in exon 7. Since position 241 is polymorphic both in *CELA3A* (p.G241A) and *CELA3B* (p.A241G), genetic analysis can directly assess whether individual variability in complex formation might alter risk for chronic pancreatitis. Here we sequenced exon 7 of *CELA3A* and *CELA3B* in a cohort of 225 subjects with chronic pancreatitis (120 alcoholic and 105 non-alcoholic) and 300 controls of Hungarian origin. Allele frequencies were 2.5% for *CELA3A* p.G241A and 1.5% for *CELA3B* p.A241G in controls, and no significant difference was observed in patients. Additionally, we identified six synonymous variants, two missense variants, a gene conversion event and ten variants in the flanking intronic regions. Variant c.643-7G>T in *CELA3B* showed an association with alcoholic chronic pancreatitis with a small protective effect (OR = 0.59, 95% CI = 0.39–0.89, *p* = 0.01). Functional analysis of missense variants revealed no major defects in secretion or activity. We conclude that variants affecting amino-acid position 241 in *CELA3A* and *CELA3B* are not associated with chronic pancreatitis, indicating that changes in complex formation between proelastases and procarboxypeptidases do not alter pancreatitis risk.

## 1. Introduction

Chronic pancreatitis (CP) is a progressive, relapsing inflammatory disorder of the pancreas which often develops in the background of genetic susceptibility [1,2,3]. Mutations in the best characterized risk genes *PRSS1* (cationic trypsinogen), *SPINK1* (pancreatic secretory trypsin inhibitor), and *CTRC* (chymotrypsin C) stimulate activation of trypsinogen and result in elevated trypsin activity in the pancreas [4,5,6,7,8,9]. More recently, loss-of-function variants in the *CPA1* gene encoding carboxypeptidase A1 were shown to increase risk for early onset CP [10]. The majority of impaired *CPA1* variants exhibited a secretion defect due to intracellular misfolding and retention. The mechanism of action of CPA1 variants was unrelated to trypsinogen activation or trypsin activity and seemed to involve endoplasmic reticulum stress as a consequence of misfolding [10]. However, it is also possible that reduced CPA1 secretion might cause predisposition to CP by other mechanisms. In this respect, we noted that procarboxypeptidase A (proCPA) often forms complexes with proelastases in the mammalian pancreas ([11] and references therein). Consequently, changes in CPA1 levels in the secretory pathway and pancreatic juice might have a significant impact on proelastases; possibly facilitating ectopic elastase activation and thereby contributing to pancreatic injury.

The human chymotrypsin-like elastase (CELA) family is encoded by five genes: *CELA1*, *CELA2A*, *CELA2B*, *CELA3A* and *CELA3B*. Due to evolutionary mutations, *CELA1* is not expressed in the human pancreas and CELA2B is an inactive protease [12,13,14]. CELA2A and CELA3B bind to proCPA1 and CELA3B also binds to proCPA2 [11,15,16,17]. Even though CELA3A is 92% identical with CELA3B in its primary structure, it does not form tight complexes with proCPA1 or proCPA2 [11]. We found that a major determinant of binding affinity was amino acid 241, which is Gly in CELA3A and Ala in CELA3B. Mutation p.G241A in CELA3A increases whereas mutation p.A241G in CELA3B reduces binding to proCPA1 [11]. Notably, position 241 is polymorphic in both elastases with minor allele frequencies of about 2% in subjects of European origin. This genetic variation should translate to individual differences in complex formation between proelastases and proCPA1. More importantly, the polymorphic nature of amino-acid 241 offers the unique opportunity to perform a genetic association study to investigate whether changes in complex formation between proelastases and procarboxypeptidases affect CP risk. To this end, in the present paper we compared allele frequencies of variants p.G241A in *CELA3A* and p.A241G in *CELA3B* between subjects with CP and controls without pancreatic disease.

## 2. Results

### 2.1. DNA Sequence Analysis of Exon 7 of Human CELA3A and CELA3B

To investigate whether changes in complex formation between human procarboxypeptidases and proelastases alter risk for CP, we investigated the frequency of variants c.722G>C (p.G241A) in *CELA3A* and c.722C>G (p.A241G) in *CELA3B* in subjects with CP and controls without pancreatic disease. We sequenced exon 7 and flanking intronic regions of *CELA3A* and *CELA3B* in 225 patients and 300 controls from the registry of the Hungarian Pancreatic Study Group. This CP cohort consisted of 120 alcoholic chronic pancreatitis (ACP) and 105 idiopathic chronic pancreatitis (ICP) patients. Sequence analysis of *CELA3A* and *CELA3B* was successfully completed for all patient samples and for 295 and 293 of the 300 control samples, respectively. In *CELA3A* we found 8 variants which included 4 intronic variants, 3 synonymous variants and 1 non-synonymous (missense) variant (Table 1). Synonymous variants c.750C>T (p.P250=) and c.753G>A (p.T251=) were found in complete linkage disequilibrium. In *CELA3B* we detected 13 variants which included 6 intronic variants, 3 synonymous variants, 3 non-synonymous (missense) variants and a gene-conversion event resulting in five nucleotide alterations that changed three amino-acids at the protein level (Table 1). Synonymous variants c.699T>C (p.H233=) and c.702C>T (p.G234=) were found in the same ACP patient.

**Figure 1:**
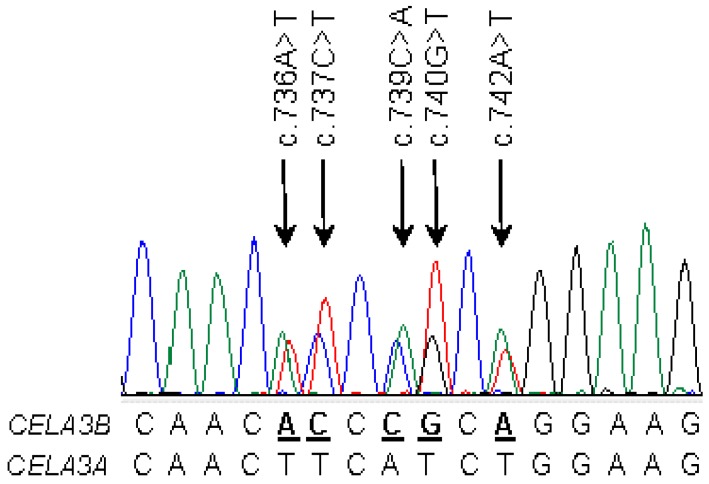
Gene conversion between *CELA3A* (donor) and *CELA3B* (acceptor). Electropherogram of *CELA3B* exon 7 in the Hungarian idiopathic chronic pancreatitis (ICP) patient. Heterozygous variations are indicated by arrows. Alignment of the relevant sequence tracts of *CELA3A* and *CELA3B* are also shown. CELA, human chymotrypsin-like elastase.

When allele frequency was considered, distribution of the variants between patients and controls showed a significant difference only for the *CELA3B* variant c.643-7G>T in intron 6, which occurred with an allele frequency of 16% in patients and 21.3% in controls (OR = 0.7; 95% CI 0.51–0.97; *p* = 0.03; Table 1). Subgroup analysis revealed that the association was driven by the ACP cohort (OR = 0.59, 95% CI = 0.39–0.89, *p* = 0.01) while it was not significant in ICP patients (*p* = 0.4; Table 2 and Table 3). Importantly, neither p.G241A in *CELA3A* nor p.A241G in *CELA3B* was significantly enriched in patients or controls. Novel missense variants identified in *CELA3B* were rare; the heterozygous c.694G>C (p.V232L) variant was found in one ACP patient and one control, whereas the heterozygous c.740G>C (p.R247P) variant was found in one control subject only. Both subjects with the p.V232L variant also carried a heterozygous c.643-7G>T *CELA3B* variant, while the subject with the p.R247P variant carried a heterozygous c.643-26C>T *CELA3B* variant. An unexpected observation in *CELA3B* was a heterozygous gene conversion event detected in a patient with ICP. A minimum of seven nucleotides in *CELA3B* between c.736 and c.742 was converted with the corresponding *CELA3A* sequence. This resulted in five nucleotide changes (c.736A>T, c.737C>T c.739C>A, c.740G>T, c.742A>T) and three amino-acid changes (p.T246F, p.R247I, p.R248W) in CELA3B (Figure 1).

Genotypes were also analyzed for common variants using dominant and recessive models but no significant differences were found between CP patients and controls (Table 4). In subgroup analysis of variant c.643-7G>T genotypes using a dominant model (GT plus TT in patients versus controls) we confirmed the underrepresentation of this variant in ACP patients (OR = 0.6, 95% CI 0.37–0.96, *p* = 0.03), whereas the same association could not be verified in the ICP group (*p* = 0.52) (Table 5 and Table 6). Using a recessive model (TT genotypes in patients versus controls) a more obvious difference was observed in the ACP group (1.7%) than in ICP patients (3.8%) versus controls (5.8%), however, due to the limited sample size it did not reach statistical significance (Table 5 and Table 6).

In summary, with the exception of the apparently protective c.643-7G>T *CELA3B* variant, we found no other association of *CELA3A* and *CELA3B* variants with CP or disease subgroups ACP and ICP. Importantly, the lack of association between variants at amino-acid position 241 and CP indicates that changes in complex formation between proelastases and procarboxypeptidases have no appreciable effect on the risk of CP.

### 2.2. Functional Analysis of Missense Variants and the Gene Conversion Event

**Figure 2:**
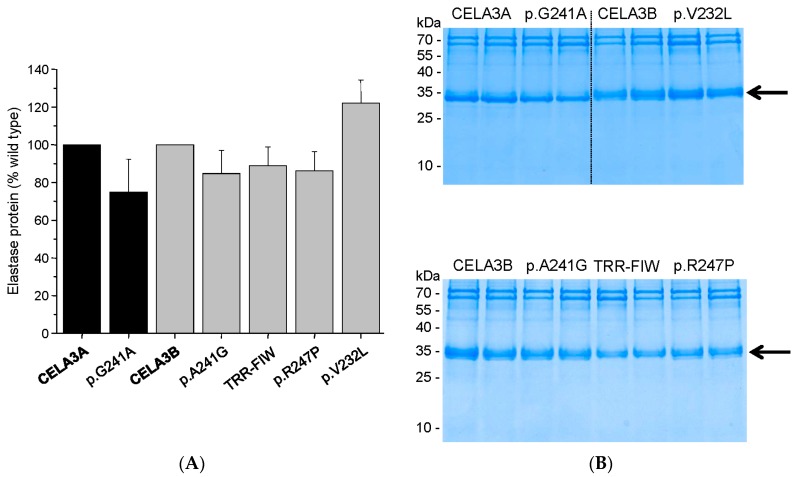
Secretion of CELA3A and CELA3B variants. Human embryonic kidney (HEK) 293T cells were transfected with the indicated constructs and conditioned medium was collected 48 h after transfection. Levels of secreted proelastases were determined by sodium dodecyl sulfate-polyacrylamide gel electrophoresis (SDS-PAGE) and Coomassie Blue staining as described in Materials and Methods. (**A**) Densitometric quantitation of secreted CELA3A and CELA3B. Average values from three transfections with standard deviation (S.D.) are indicated; expressed as percent of wild-type levels; (**B**) Representative gels are shown with samples loaded in duplicate. The molecular mass markers were PageRuler Prestained Protein Ladder (Thermo Scientific, Waltham, MA, USA; PI-26616). The arrow indicates the proelastase bands. The diffuse appearance of the CELA3B bands is likely due to *N*-glycosylation. TRR-FIW designates the p.T246F, p.R247I, p.R248W variant which is the product of the gene conversion.

**Figure 3:**
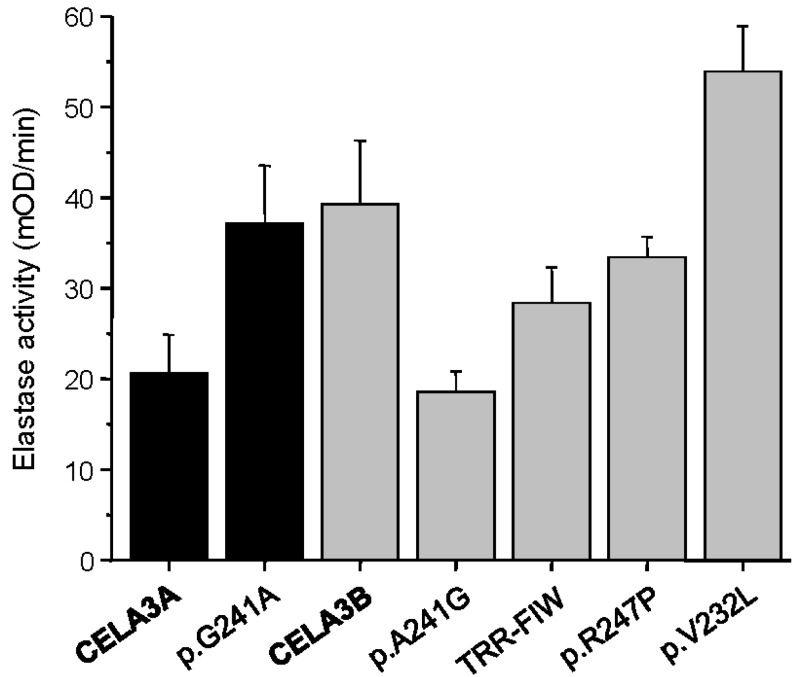
Activity of CELA3A and CELA3B variants in the conditioned medium of transfected HEK 293T cells. Proelastases were activated with trypsin and elastase activity was determined as described in Materials and Methods. Average values ± S.D. from three transfections are shown. TRR-FIW designates the p.T246F, p.R247I, p.R248W variant which is the product of the gene conversion.

To characterize how missense mutations in *CELA3A* and *CELA3B* and the gene conversion in CELA3B alter elastase secretion and activity, we transfected human embryonic kidney (HEK) 293T cells with expression plasmids and measured proelastase levels in the conditioned medium by sodium dodecyl sulfate-polyacrylamide gel electrophoresis (SDS-PAGE) and elastase activity by enzymatic assays. When elastase secretion into the medium was determined at 48 h after transfection, all variants were secreted normally, as judged by Coomassie Blue staining and densitometry of stained gels (Figure 2). *CELA3B* variant p.V232L exhibited a slight (~20%) but reproducible increase in secretion. Similarly, elastase activity of the conditioned medium after activation with trypsin showed no major differences among the variants and wild-type enzymes when measured with the Suc-Ala-Ala-Pro-Ala-*p*-nitroanilide chromogenic peptide substrate (Figure 3). Consistent with its increased levels of secretion, variant p.V232L exhibited higher activity while the activity of the gene conversion variant (p.T246F, p.R247I, p.R248W) was slightly (1.4-fold) reduced relative to wild-type CELA3B. Interestingly, variants p.G241A in CELA3A and p.A241G in CELA3B had opposite effects on elastase activity. Thus, activity of the CELA3A p.G241A variant was increased by 1.8-fold, whereas activity of the CELA3B p.A241G variant was decreased by 2.2-fold relative to their respective wild-type controls. Even though these changes in activity controlled by the amino acid at position 241 (Gly versus Ala) are intriguing mechanistically, they have no impact on CP risk as demonstrated by the lack of genetic association described above.

## 3. Discussion

In the present study we tested the hypothesis that changes in complex formation between human proelastases and procarboxypeptidases might alter risk for CP. We speculated that individual variations in the levels of these zymogen complexes may influence digestive enzyme activation as the free, uncomplexed enzyme should be more susceptible to activation than its complexed form. Indeed, we recently demonstrated that activation of human procarboxypeptidases A1 and A2 were delayed when bound to human proCELA3B [11]. Although activation of proCELA3B did not seem to be directly affected by complex formation, changes in folding, solubility, packaging and secretion might be altered which can ultimately promote ectopic elastase activation inside the pancreas. To identify whether such mechanisms play a role in CP risk, we took advantage of the observation that the primary amino-acid determinants of complex formation in CELA3A and CELA3B are polymorphic in the population. Thus, normally proCELA3A binds poorly to procarboxypeptidases due to the presence of Gly241, whereas proCELA3B which contains Ala241 forms tight complexes [11]. Individuals carrying a heterozygous p.G241A mutation in CELA3A should have increased complex levels in their pancreatic secretions. Conversely, subjects with the p.A241G variant in CELA3B should exhibit decreased complex formation and higher levels of free zymogens. Should CP risk be elevated by decreased complex formation, one would expect to observe underrepresentation of the CELA3A p.G241A variant and/or enrichment of the CELA3B p.A241G variant in a cohort of CP patients versus population controls.

To determine allele frequencies of these variants, we sequenced exon 7 and flanking intronic regions of *CELA3A* and *CELA3B* in CP patients and controls of Hungarian origin. We found that allele frequencies of *CELA3A* p.G241A and *CELA3B* p.A241G were not significantly different between the two groups, indicating that these variants do not alter CP risk. Despite the lack of statistical significance, an opposing trend for enrichment of the two variants was apparent. Thus, *CELA3A* variant p.G241A was slightly overrepresented in controls while *CELA3B* variant p.A241G was overrepresented in patients. Assuming the OR values represent the true effect sizes, we would need a much larger cohort to confirm these findings with statistical significance. These effects, on the other hand, would be still clinically insignificant, which does not justify further extension of the studies.

In addition to the position 241 variants discussed above, we identified six synonymous variants, two missense variants, a gene conversion event and ten variants in the flanking intronic regions. Intriguingly, *CELA3B* variant c.643-7G>T in intron 6 was significantly underrepresented in ACP patients, indicating an approximately 1.7-fold protective effect against CP. This variant is located relatively close to the pre-mRNA splice site and it is conceivable that it might disrupt splicing and decrease *CELA3B* expression. This finding suggests that elastase activity contributes to pancreatitis risk and warrants extension of sequence analysis of the *CELA3A* and *CELA3B* genes and replication of the observed association(s) in an independent cohort. None of the other variants showed a significant difference in frequency between CP patients and controls. Finally, we performed functional analysis on all missense variants detected in this study and found no major defects in proelastase secretion or elastase activity.

We discovered a rare gene conversion event between exons 7 of *CELA3A* and *CELA3B* in an individual with ICP. Gene conversion is a non-reciprocal exchange of genetic information between homologous DNA sequences, most likely as a result of mismatch repair following a heteroduplex formation between the donor and acceptor genes [18]. DNA exchange by gene conversion is of paramount importance for the evolution of gene families but it can also cause human diseases [18] including CP and other pancreatic pathologies. Gene conversion events between *PRSS1* and *PRSS2* or between *PRSS1* and the pseudogene *PRSS3P2* were shown to generate pathogenic alleles that cause hereditary pancreatitis [19,20,21]. More recently, a recombination allele of the carboxyl ester lipase gene (*CEL*) and its pseudogene *CELP* was described as a novel genetic risk factor for CP [22]. Finally, conversion events between the *SBDS* gene and its paralogous duplicated pseudogene *SBDSP* cause Shwachman-Bodian-Diamond syndrome, an autosomal recessive disorder with pancreatic exocrine insufficiency, hematologic dysfunction, and skeletal abnormalities [23]. The newly found gene conversion event between *CELA3A* and *CELA3B* was detected in a CP patient, however, this is likely an accidental finding as we found no functional defect with respect to secretion or activity of the converted CELA3B that might suggest a pathogenic role in CP.

In summary, our study demonstrated that variants affecting amino-acid position 241 in human *CELA3A* and *CELA3B* are not associated with CP, indicating that changes in complex formation between human proelastases and procarboxypeptidases do not influence the risk for CP. The observation that intronic variant c.643-7G>T in *CELA3B* was significantly underrepresented in ACP patients suggests this might be a protective variant.

## 4. Materials and Methods

### 4.1. Nomenclature

Nucleotide numbering reflects coding DNA numbering with +1 corresponding to the A of the ATG translation initiation codon in the *CELA3A* and *CELA3B* reference sequences (*Homo sapiens* chromosome 1, GRCh38.p2 primary assembly, NC_000001.11). Note that the reference sequence for *CELA3A* contains the minor alleles at positions c.722 (p.241) and c.795+21. Similarly, in *CELA3B* positions c.780 (p.260) and c.795+21 represent minor alleles. To better contrast position 241 between the two genes, we used the major c.722G allele (p.G241) as a reference for *CELA3A* and designated the variant at this position as c.722G>C (p.G241A). Amino-acid variants are numbered starting with the initiator methionine of the primary translation products of *CELA3A* and *CELA3B*.

### 4.2. Study Subjects

De-identified genomic DNA samples were obtained from the registry of the Hungarian Pancreatic Study Group (ethical approval: TUKEB 22254-1/2012/EKU, biobanking approval: IF702-19/2012). Individuals in the registry were recruited from 11 Hungarian centers between 2012 and 2016 and all gave informed consent according to the ethical guidelines of the Declaration of Helsinki. The study was approved by the Institutional Review Board at Boston University (“Analysis of susceptibility genes in patients with chronic pancreatitis”; IRB number H-35382). A total of 225 unrelated patients with CP, including 120 with alcoholic CP (ACP) and 105 with idiopathic CP (ICP) and 300 control subjects with no pancreatic disease were studied. Sequence analysis of *CELA3A* and *CELA3B* was successfully completed for 295 and 293 of the 300 control samples, respectively. Patient characteristics are summarized in Table 7. Diagnosis of CP was based on two or more of the following findings: history of recurrent acute pancreatitis, pancreatic calcification detected by CT or ultrasonography, and pancreatic ductal irregularities on endoscopic retrograde cholangiopancreatography (ERCP) or magnetic resonance cholangiopancreatography (MRCP) examinations. ACP was diagnosed when the patient history included alcohol consumption of more than 80 g/day (men) or 60 g/day (women) for at least two years. ICP was diagnosed in the absence of precipitating or risk factors for pancreatitis, such as alcohol abuse, medications, trauma, metabolic disorders or infection. A representative sample of this cohort including 100 patients (49 ACP and 51 ICP) and 100 controls was previously characterized for the clinically common *SPINK1* variants; the p.N34S variant was found in three ICP patients and in one control subject while the c.194+2T>C variant was detected in two ACP and one ICP patients but not in controls [24].

### 4.3. DNA Sequencing

Primers were designed against intronic sequences flanking exon 7 in *CELA3A* and *CELA3B*. Primer sequences and amplicon sizes are given in Table 8. Polymerase chain reactions (PCR) were performed using 0.75 U HotStar Taq DNA polymerase (Qiagen, Valencia, CA, USA), 0.2 mM dNTP, 5 μL 5× Q-solution and 2.5 µL 10× PCR buffer (Qiagen), 0.5 μM primers, and 10–50 ng genomic DNA template in a volume of 25 μL. PCR reactions were started by a 15-min initial heat activation at 95 °C followed by 35 cycles of 30 s denaturation at 94 °C, 30-s annealing at 55 °C, and 40 s extension at 72 °C; and finished by a final extension for 5 min at 72 °C. Products of all PCR reactions were verified by 2% agarose gel electrophoresis. The PCR amplicons (5 µL) were treated with 1 µL FastAP Thermosensitive Alkaline Phosphatase and 0.5 µL Exonuclease I (Thermo Fisher Scientific, Waltham, MA, USA) for 15 min at 37 °C and the reaction was stopped by heating the samples to 85 °C for 15 min. Sanger sequencing was performed using the reverse PCR primer as sequencing primer. Amplicons containing heterozygous deletion variants in intron 6 of *CELA3A* were also sequenced with the forward PCR primer. New variants were confirmed by a second independent PCR amplification and also by sequencing the other DNA strand.

### 4.4. Plasmid Construction and Mutagenesis

Construction of expression plasmids for human *CELA3A* and *CELA3B* carrying C-terminal His tags in the pcDNA3.1(−) vector was described previously [11]. Mutations were introduced by overlap extension PCR mutagenesis.

### 4.5. Cell Culture and Transfection

Human embryonic kidney (HEK) 293T cells were cultured in 6-well tissue culture plates at a density of 10^6^ cells per well in Dulbecco’s Modified Eagle Medium (DMEM, Thermo Fisher Scientific) supplemented with 10% fetal bovine serum, 4 mM glutamine, and 1% penicillin/streptomycin at 37 °C in a humidified atmosphere containing 5% CO_2_. Transfections were performed using 4 μg expression plasmid and 10 μL Lipofectamine 2000 (Invitrogen, Carlsbad, CA, USA) in 2 mL DMEM. After overnight incubation at 37 °C, cells were rinsed and overlaid with 2 mL Opti-MEM reduced serum medium (Gibco). Media and cells were collected 48 h after this medium change.

### 4.6. Measurement of Elastase Activity in Conditioned Media

Aliquots (100 μL) of conditioned media were supplemented with 0.1 M Tris-HCl (pH 8.0) by adding 10 μL from a 1 M stock solution and 1 mM calcium by adding 1 μL of a 0.1 M CaCl_2_ solution. Proelastases were activated with 100 nM human cationic trypsin (final concentration) for 30 min at 37 °C. Aliquots (20 μL) of activated elastases were then mixed with 175 μL assay buffer (0.1 M Tris-HCl (pH 8.0), 1 mM CaCl_2_, 0.05% Tween 20) and elastase activity was measured by adding 5 μL of 6 mM Suc-Ala-Ala-Pro-Ala-*p*-nitroanilide substrate. The release of the yellow *p*-nitroaniline was followed at 405 nm in a SpectraMax Plus384 microplate reader (Molecular Devices, Sunnyvale, CA, USA) for 5 min. Rates of substrate cleavage were calculated from fits to the initial linear portions of the curves and expressed in milliOD/min units.

### 4.7. SDS-PAGE

Conditioned media (180 μL) were precipitated with 10% trichloroacetic acid (final concentration); the precipitate was collected by centrifugation and resuspended in 20 μL Laemmli sample buffer containing 100 mM dithiothreitol, heat-denatured at 95 °C for 5 min and run on 15% SDS-polyacrylamide gels. The gels were stained with Coomassie Blue R-250. Densitometric quantitation of bands was carried out with the Gel Doc XR+ gel documentation system and Image Lab software (Bio-Rad, Hercules, CA, USA).

## Figures and Tables

**Table 1:** Allele frequency of *CELA3A* and *CELA3B* variants in patients with chronic pancreatitis and controls without pancreatic disease. Variants affecting position 241 are highlighted in bold. OR, odds ratio; CI, confidence interval. Asterisk indicates significant association.

***CELA3A***	**Nucleotide Change**	**Amino Acid Change**	**Patient Alleles**	**Control Alleles**	**OR**	***p* Value**	**95% CI**
Intron 6	c.643-78T>C	–	81/450 (18%)	124/590 (21%)	0.83	0.23	0.60–1.13
Intron 6	c.643-17delC	–	21/450 (4.7%)	24/590 (4.1%)	1.15	0.64	0.63–2.1
Intron 6	c.643-13_12delCT	–	3/450 (0.7%)	5/590 (0.8%)	0.79	0.74	0.19–3.3
Exon 7	c.699C>T	p.H233=	1/450 (0.2%)	0/590 (0%)	–	–	–
**Exon 7**	**c.722G>C**	**p.G241A**	**9/450 (2%)**	**15/590 (2.5%)**	**0.78**	**0.57**	**0.34–1.8**
Exon 7	c.750C>T	p.P250=	105/450 (23.3%)	157/590 (26.6%)	0.84	0.23	0.63–1.12
Exon 7	c.753G>A	p.T251=	105/450 (23.3%)	157/590 (26.6%)	0.84	0.23	0.63–1.12
Intron 7	c.795+21C>A		321/450 (71.3%)	399/590 (67.6%)	1.19	0.2	0.91–1.56
***CELA3B***	**Nucleotide Change**	**Amino Acid Change**	**Patient Alleles**	**Control Alleles**	**OR**	***p* Value**	**95% CI**
Intron 6	c.643-26C>T	–	20/450 (4.4%)	25/586 (4.3%)	1.04	0.89	0.57–1.91
Intron 6	c.643-9C>T	–	2/450 (0.4%)	0/586 (0%)	–	–	–
Intron 6	c.643-7G>T	–	72/450 (16%)	125/586 (21.3%)	0.7	0.03 *	0.51–0.97
Exon 7	c.694G>C	p.V232L	1/450 (0.2%)	1/586 (0.2%)	1.3	0.85	0.08–20.88
Exon 7	c.699T>C	p.H233=	1/450 (0.2%)	0/586 (0%)	–	–	–
Exon 7	c.702C>T	p.G234=	1/450 (0.2%)	0/586 (0%)	–	–	–
**Exon 7**	**c.722C>G**	**p.A241G**	**10/450 (2.2%)**	**9/586 (1.5%)**	**1.46**	**0.42**	**0.59–3.61**
Exon 7	c.736A>T, c.737C>T, c.739C>A, c.740G>T, c.742A>T	p.T246F, p.R247I, p.R248W	1/450 (0.2%)	0/586 (0%)	–	–	–
Exon 7	c.740G>C	p.R247P	0/450 (0%)	1/586 (0.2%)	–	–	–
Exon 7	c.780T>C	p.I260=	445/450 (98.9%)	580/586 (99%)	0.92	0.89	0.28–3.03
Intron 7	c.795+21C>A	–	340/450 (75.6%)	413/586 (70.5%)	1.29	0.07	0.97–1.71
Intron 7	c.795+22G>A	–	1/450 (0.2%)	1/586 (0.2%)	1.3	0.85	0.08–20.88
Intron 7	c.795+73G>A	–	0/450 (0%)	1/586 (0.2%)	–	–	–

**Table 2:** Allele frequency of *CELA3A* and *CELA3B* variants in patients with alcoholic chronic pancreatitis (ACP) and controls without pancreatic disease. Variants affecting position 241 are highlighted in bold. OR, odds ratio; CI, confidence interval. Asterisk indicates significant association.

***CELA3A***	**Nucleotide Change**	**Amino Acid Change**	**ACP Patient Alleles**	**Control Alleles**	**OR**	***p* Value**	**95% CI**
Intron 6	c.643-78T>C	–	43/240 (17.9%)	124/590 (21%)	0.82	0.31	0.56–1.2
Intron 6	c.643-17delC	–	13/240 (5.4%)	24/590 (4.1%)	1.35	0.4	0.68–2.7
Intron 6	c.643-13_12delCT	–	1/240 (0.4%)	5/590 (0.8%)	0.49	0.52	0.06–4.21
**Exon 7**	**c.722G>C**	**p.G241A**	**4/240 (1.7%)**	**15/590 (2.5%)**	**0.65**	**0.45**	**0.21–1.98**
Exon 7	c.750C>T	p.P250=	57/240 (23.8%)	157/590 (26.6%)	0.86	0.4	0.60–1.22
Exon 7	c.753G>A	p.T251=	57/240 (23.8%)	157/590 (26.6%)	0.86	0.4	0.60–1.22
Intron 7	c.795+21C>A	–	167/240 (69.6%)	399/590 (67.6%)	1.1	0.58	0.79–1.51
***CELA3B***	**Nucleotide Change**	**Amino Acid Change**	**ACP Patient Alleles**	**Control Alleles**	**OR**	***p* Value**	**95% CI**
Intron 6	c.643-26C>T	–	13/240 (5.4%)	25/586 (4.3%)	1.29	0.48	0.65–2.56
Intron 6	c.643-9C>T	–	2/240 (0.8%)	0/586 (0%)	–	–	–
Intron 6	c.643-7G>T	–	33/240 (13.8%)	125/586 (21.3%)	0.59	0.01 *	0.39–0.89
Exon 7	c.694G>C	p.V232L	1/240 (0.4%)	1/586 (0.2%)	2.45	0.53	0.15–39.29
Exon 7	c.699T>C	p.H233=	1/240 (0.4%)	0/586 (0%)	–	–	–
Exon 7	c.702C>T	p.G234=	1/240 (0.4%)	0/586 (0%)	–	–	–
**Exon 7**	**c.722C>G**	**p.A241G**	**5/240 (2.1%)**	**9/586 (1.5%)**	**1.37**	**0.58**	**0.45–4.11**
Exon 7	c.780T>C	p.I260=	237/240 (98.8%)	580/586 (99%)	0.82	0.78	0.20–3.29
Intron 7	c.795+21C>A	–	179/240 (74.6%)	413/586 (70.5%)	1.23	0.24	0.88–1.73
Intron 7	c.795+22G>A	–	1/240 (0.4%)	1/586 (0.2%)	2.45	0.53	0.15–39.29

**Table 3:** Allele frequency of *CELA3A* and *CELA3B* variants in patients with idiopathic chronic pancreatitis (ICP) and controls without pancreatic disease. Variants affecting position 241 are highlighted in bold. OR, odds ratio; CI, confidence interval.

***CELA3A***	**Nucleotide Change**	**Amino Acid Change**	**ICP Patient Alleles**	**Control Alleles**	**OR**	***p* Value**	**95% CI**
Intron 6	c.643-78T>C	–	38/210 (18.1%)	124/590 (21%)	0.83	0.37	0.56–1.24
Intron 6	c.643-17delC	–	8/210 (3.8%)	24/590 (4.1%)	0.93	0.87	0.42–2.11
Intron 6	c.643-13_12delCT	–	2/210 (1%)	5/590 (0.8%)	1.13	0.89	0.22–5.84
Exon 7	c.699C>T	p.H233=	1/210 (0.5%)	0/590 (0%)	–	–	–
**Exon 7**	**c.722G>C**	**p.G241A**	**5/210 (2.4%)**	**15/590 (2.5%)**	**0.93**	**0.89**	**0.34–2.61**
Exon 7	c.750C>T	p.P250=	48/210 (22.9%)	157/590 (26.6%)	0.82	0.29	0.56–1.18
Exon 7	c.753G>A	p.T251=	48/210 (22.9%)	157/590 (26.6%)	0.82	0.29	0.56–1.18
Intron 7	c.795+21C>A	–	154/210 (73.3%)	399/590 (67.6%)	1.32	0.13	0.93–1.87
***CELA3B***	**Nucleotide Change**	**Amino Acid Change**	**ICP Patient Alleles**	**Control Alleles**	**OR**	***p* Value**	**95% CI**
Intron 6	c.643-26C>T	–	7/210 (3.3%)	25/586 (4.3%)	0.77	0.56	0.33–1.82
Intron 6	c.643-7G>T	–	39/210 (18.6%)	125/586 (21.3%)	0.84	0.4	0.56–1.26
**Exon 7**	**c.722C>G**	**p.A241G**	**5/210 (2.4%)**	**9/586 (1.5%)**	**1.56**	**0.43**	**0.52–4.72**
Exon 7	c.736A>T, c.737C>T, c.739C>A, c.740G>T, c.742A>T	p.T246F, p.R247I, p.R248W	1/210 (0.5%)	0/586 (0%)	–	–	–
Exon 7	c.780T>C	p.I260=	208/210 (99%)	580/586 (99%)	1.08	0.93	0.22–5.37
Intron 7	c.795+21C>A	–	161/210 (76.7%)	413/586 (70.5%)	1.38	0.09	0.96–1.98

**Table 4:** Genotype distribution of common *CELA3A* and *CELA3B* variants. Results were analyzed assuming dominant (shown in italics) or recessive models of inheritance. OR, odds ratio; CI, confidence interval.

***CELA3A***	**Nucleotide Change**	**Genotype**	**Patients**	**Controls**	**OR**	***p* Value**	**95% CI**
Intron 6	c.643-78T>C	TT	153/225 (68%)	186/295 (63%)	–	–	–
		TC	63/225 (28%)	94/295 (31.9%)	*0.80*	*0.25*	*0.56–1.16*
		CC	9/225 (4%)	15/295 (5.1%)	0.78	0.56	0.33–1.81
Exon 7	c.750C > T	CC	132/225 (58.7%)	158/295 (53.5%)	–	–	–
		CT	81/225 (36%)	117/295 (39.7%)	*0.81*	*0.25*	*0.57–1.15*
		TT	12/225 (5.3%)	20/295 (6.8%)	0.77	0.5	0.37–1.62
Exon 7	c.753G > A	GG	132/225 (58.7%)	158/295 (53.5%)	–	–	–
		GA	81/225 (36%)	117/295 (39.7%)	*0.81*	*0.25*	*0.57–1.15*
		AA	12/225 (5.3%)	20/295 (6.8%)	0.77	0.5	0.37–1.62
Intron 7	c.795+21C>A	CC	17/225 (7.6%)	32/295 (10.8%)	–	–	–
		CA	95/225 (42.2%)	127/295 (43.1%)	*1.49*	*0.21*	*0.80–2.76*
		AA	113/225 (50.2%)	136/295 (46.1%)	1.18	0.35	0.83–1.67
***CELA3B***	**Nucleotide Change**	**Genotype**	**Patients**	**Controls**	**OR**	***p* Value**	**95% CI**
Intron 6	c.643-7G>T	GG	159/225 (70.6%)	185/293 (63.1%)	–	–	–
		GT	60/225 (26.7%)	91/293 (31.1%)	*0.71*	*0.07*	*0.49–1.03*
		TT	6/225 (2.7%)	17/293 (5.8%)	0.44	0.09	0.17–1.15
Exon 7	c.780T>C	TT	1/225 (0.5%)	0/293 (0%)	–	–	–
		TC	3/225 (1.3%)	6/293 (2%)	–	–	–
		CC	221/225 (98.2%)	287/293 (98%)	1.15	0.83	0.32–4.14
Intron 7	c.795+21C>A	CC	10/225 (4.4%)	24/293 (8.2%)	–	–	–
		CA	90/225 (40%)	125/293 (42.7%)	*1.92*	*0.09*	*0.90–4.1*
		AA	125/225 (55.6%)	144/293 (49.1%)	1.29	0.15	0.91–1.8

**Table 5:** Genotype distribution of common *CELA3A* and *CELA3B* variants in patients with alcoholic chronic pancreatitis (ACP) and controls without pancreatic disease. Results were analyzed assuming dominant (shown in italics) or recessive models of inheritance. OR, odds ratio; CI, confidence interval. Asterisk indicates significant association.

***CELA3A***	**Nucleotide Change**	**Genotype**	**ACP Patients**	**Controls**	**OR**	***p* Value**	**95% CI**
Intron 6	c.643-78T>C	TT	83/120 (69.2%)	186/295 (63%)	–	–	–
		TC	31/120 (25.8%)	94/295 (31.9%)	*0.76*	*0.24*	*0.48–1.2*
		CC	6/120 (5%)	15/295 (5.1%)	0.98	0.97	0.37–2.6
Exon 7	c.750C>T	CC	70/120 (58.3%)	158/295 (53.5%)	–	–	–
		CT	43/120 (35.8%)	117/295 (39.7%)	*0.82*	*0.38*	*0.54–1.27*
		TT	7/120 (5.9%)	20/295 (6.8%)	0.85	0.72	0.35–2.07
Exon 7	c.753G>A	GG	70/120 (58.3%)	158/295 (53.5%)	–	–	–
		GA	43/120 (35.8%)	117/295 (39.7%)	*0.82*	*0.38*	*0.54–1.27*
		AA	7/120 (5.9%)	20/295 (6.8%)	0.85	0.72	0.35–2.07
Intron 7	c.795+21C>A	CC	10/120 (8.3%)	32/295 (10.8%)	–	–	–
		CA	53/120 (44.2%)	127/295 (43.1%)	*1.34*	*0.44*	*0.64–2.82*
		AA	57/120 (47.5%)	136/295 (46.1%)	1.06	0.8	0.69–1.62
***CELA3B***	**Nucleotide Change**	**Genotype**	**ACP Patients**	**Controls**	**OR**	***p* Value**	**95% CI**
Intron 6	c.643-7G>T	GG	89/120 (74.1%)	185/293 (63.1%)	–	–	–
		GT	29/120 (24.2%)	91/293 (31.1%)	*0.6*	0.03 *	*0.37–0.96*
		TT	2/120 (1.7%)	17/293 (5.8%)	0.28	0.09	0.06–1.21
Exon 7	c.780T>C	TT	1/120 (0.8%)	0/293 (0%)	–	–	–
		TC	1/120 (0.8%)	6/293 (2%)	–	–	–
		CC	118/120 (98.4%)	287/293 (98%)	1.23	0.8	0.25–6.2
Intron 7	c.795+21C>A	CC	7/120 (5.8%)	24/293 (8.2%)	–	–	–
		CA	47/120 (39.2%)	125/293 (42.7%)	*1.44*	*0.41*	*0.60–3.44*
		AA	66/120 (55%)	144/293 (49.1%)	1.27	0.28	0.83–1.94

**Table 6:** Genotype distribution of common *CELA3A* and *CELA3B* variants in patients with idiopathic chronic pancreatitis (ICP). Results were analyzed assuming dominant (shown in italics) or recessive models of inheritance. OR, odds ratio; CI, confidence interval.

***CELA3A***	**Nucleotide Change**	**Genotype**	**ICP Patients**	**Controls**	**OR**	***p* Value**	**95% CI**
Intron 6	c.643-78T>C	TT	70/105 (66.6%)	186/295 (63%)	–	–	–
		TC	32/105 (30.5%)	94/295 (31.9%)	*0.85*	*0.51*	*0.53–1.36*
		CC	3/105 (2.9%)	15/295 (5.1%)	0.55	0.35	0.16–1.94
Exon 7	c.750C>T	CC	62/105 (59%)	158/295 (53.5%)	–	–	–
		CT	38/105 (36.2%)	117/295 (39.7%)	*0.8*	*0.33*	*0.51–1.26*
		TT	5/105 (4.8%)	20/295 (6.8%)	0.69	0.47	0.25–1.88
Exon 7	c.753G>A	GG	62/105 (59%)	158/295 (53.5%)	–	–	–
		GA	38/105 (36.2%)	117/295 (39.7%)	*0.8*	*0.33*	*0.51–1.26*
		AA	5/105 (4.8%)	20/295 (6.8%)	0.69	0.47	0.25–1.88
Intron 7	c.795+21C>A	CC	7/105 (6.7%)	32/295 (10.8%)	–	–	–
		CA	42/105 (40%)	127/295 (43.1%)	*1.7*	*0.22*	*0.73–3.99*
		AA	56/105 (53.3%)	136/295 (46.1%)	1.34	0.2	0.85–2.09
***CELA3B***	**Nucleotide Change**	**Genotype**	**ICP Patients**	**Controls**	**OR**	***p* Value**	**95% CI**
Intron 6	c.643-7G>T	GG	70/105 (66.7%)	185/293 (63.1%)	–	–	–
		GT	31/105 (29.5%)	91/293 (31.1%)	*0.86*	*0.52*	*0.54–1.37*
		TT	4/105 (3.8%)	17/293 (5.8%)	0.64	0.44	0.21–1.96
Exon 7	c.780T>C	TT	0/105 (0%)	0/293 (0%)	–	–	–
		TC	2/105 (1.9%)	6/293 (2%)	–	–	–
		CC	103/105 (98.1%)	287/293 (98%)	1.08	0.93	0.21–5.42
Intron 7	c.795+21C>A	CC	3/105 (2.8%)	24/293 (8.2%)	–	–	–
		CA	43/105 (41%)	125/293 (42.7%)	*3.03*	*0.08*	*0.89–10.29*
		AA	59/105 (56.2%)	144/293 (49.1%)	1.33	0.22	0.85–2.08

**Table 7:** Study population. Age represents age at recruitment. Mean ± S.D. are shown. CP: chronic pancreatitis.

	ACP *n* = 120		ICP *n* = 105		All CP *n* = 225		Controls *n* = 300	
	Male	Female	Male	Female	Male	Female	Male	Female
	107	13	63	42	170	55	180	120
Age	56 ± 9	51 ± 8	58 ± 12	61 ± 14	57 ± 11	59 ± 14	52 ± 11	52 ± 13
Age of onset	47 ± 11	46 ± 9	49 ± 12	52 ± 17	48 ± 11	50 ± 16	–	–

**Table 8:** Oligonucleotide primers used for PCR amplification of exon 7 in *CELA3A* and *CELA3B*.

	Primer Sequence (5′ → 3′)	Amplicon	Annealing Temperature
CELA3A x7 Forward	TCA GAG GTG TCA AGT AAT GTC AG	461 bp	55 °C
CELA3A x7 Reverse	CTT GAT GGC TTC TGG GTG G		
CELA3B x7 Forward	TCA GAG GAG TCA GGT AAT GTC G	484 bp	55 °C
CELA3B x7 Reverse	AAG TTC AGC TGT AGT TCC AAG C		
